# Protected surface state in stepped Fe (0 18 1)

**DOI:** 10.1038/s41598-017-06896-4

**Published:** 2017-07-26

**Authors:** Manuel Izquierdo, Piero Torelli, Jun Fujii, Giancarlo Panaccione, Ivana Vobornik, Giorgio Rossi, Fausto Sirotti

**Affiliations:** 1 European XFEL GmbH, Holzkoppel 4, 22869 Schenefeld, Germany; 2 Synchrotron Soleil, L’Orme des Merisiers St Aubin, BP 48, 91192 Gif-sur-Yvette, France; 3 CNR-IOM, TASC Laboratory, in Area Science Park, S.S.14, Km 163.5, I-34149 Trieste, Italy; 4 Dipartimento di Fisica, Università di Milano, via Celoria 16, 20133 Milano, Italy; 5 Laboratoire de Physique de la Matière Condensée UMR 7643, CNRS, Ecole Polytechnique, Université Paris Saclay, 91128 Palaiseau, France

## Abstract

Carbon (C) surface segregation from bulk stabilizes the Fe(0 18 1) vicinal surface by forming a c(3$\sqrt{2}$ × $\sqrt{2}$ reconstruction with C zig-zag chains oriented at 45° with respect to the iron surface steps. The iron surface electronic states as measured by high resolution ARPES at normal emission with polarized synchrotron radiation split in two peaks that follow distinct energy dispersion curves. One peak follows the dispersion of the carbon superstructure and is photoexcited only when the polarization vector is parallel to the steps, the second peak disperses similarly to the pristine Fe(0 0 1) surface. Such surface electronic structure is robust as it persists even after coating with an Ag overlayer. The robustness of this surface electronic structure and its similarity with that of the clean Fe(0 0 1) surface make this system of interest for magnetic and spintronic properties such as magneto tunnel junctions based on Fe/MgO interface.

## Introduction

The magnetic properties of materials can be drastically modified upon reducing dimensionality. The lowest realized dimensionality, atomic magnetism on surfaces, represents the limit of magnetic anisotropy at cryogenic temperatures^1^. One dimensional (1D) magnetism was realized using stepped surfaces, even in non-magnetic semiconductors^2, 3^. Control of the magnetic anisotropy of magnetic materials has been realized using stepped surfaces. Using this route, 1D chains with perpendicular magnetic anisotropy^4^ (PMA) or exotic PMA in Fe(110) ultrathin films on stepped W(110) were produced^5^. Nevertheless, little is known about the modification of magnetic surface states on stepped terraces, probably due to the fragility of the clean surfaces against adsorption of overlayers (typically, partial surface oxidation suppresses surface states). Most of the studies were performed with scanning tunneling microscopy^6–12^. Monoatomic steps of Fe films on Au display an enhancement of intensity at the step edges. It has been understood as scattering of surface state (SS) electrons by step edges^6, 10^. Scattering effects have been used to partially explain interlayer exchange coupling and tunnel magnetoresistance. In this context, magnetic surface states can impact the performance of Fe/Ag/Fe spin valves^13, 14^ or Fe/MgO/Fe magneto-tunnel junctions (MTJ). The high magnetoresistance depends on the electronic properties at the interfaces^15–17^. In both examples passivation of the outermost layer is required. In the latter case, intercalation of a carbon (C) layer at the Fe-MgO interface has shown to improve the performance of the junctions^18, 19^. The observed behavior can be understood assuming that carbon passivates the surface and as a consequence it prevents the intermixing and oxidation of Fe with the MgO. Indeed, evidences of passivation of the Fe(0 0 1) surface by carbon have been observed at the C(c(3$\surd$2 × $\surd$2)):Fe(0 0 1) interface^20^. In this particular termination, the carbon forms zigzag chains with one dimensional surface states^21^.

Here we present our results on the carbon terminated iron surfaces when confinement effects are introduced by the regular steps of a vicinal surface. Our multitechnique approach shows that introducing a regular arrays of steps on a C(c(3$\surd$2 × $\surd$2)):Fe(0 0 1) surface results in two effects: passivation of the interface and stabilization of the iron surface states with its high spin SS polarization. Preserving or restoring a surface state by surface engineering has never been reported before. It indicates a new possible route for realizing 2D protected states as it is the case of interface 2D electron gas in semiconductor heterojunctions or in topological insulators. Furthermore, it can be used to optimize Fe based devices like spin valves and tunnel junctions.

## Results

The Fe(0 18 1) surface termination of the sample was obtained from a Fe single crystal cut with an offset angle of 2.7° from the Fe(0 0 1) with the steps aligned parallel to the [1 0 0] direction 1(a). The choice of this surface was done based on the fact that among all the low index surfaces of Fe, extensively studied^20, 22–26^, the (0 0 1) exhibits the largest spin polarization of the surface states^22–24, 27–31^. A miscut of 2.7° results in terraces that determine lateral confinement of the surface states^32, 33^. The terraces have their normal parallel to the [0 0 1] direction and, as a consequence, the surface states are those well known of the Fe(0 0 1) surface, but modified by lateral confinement.

**Figure 1:**
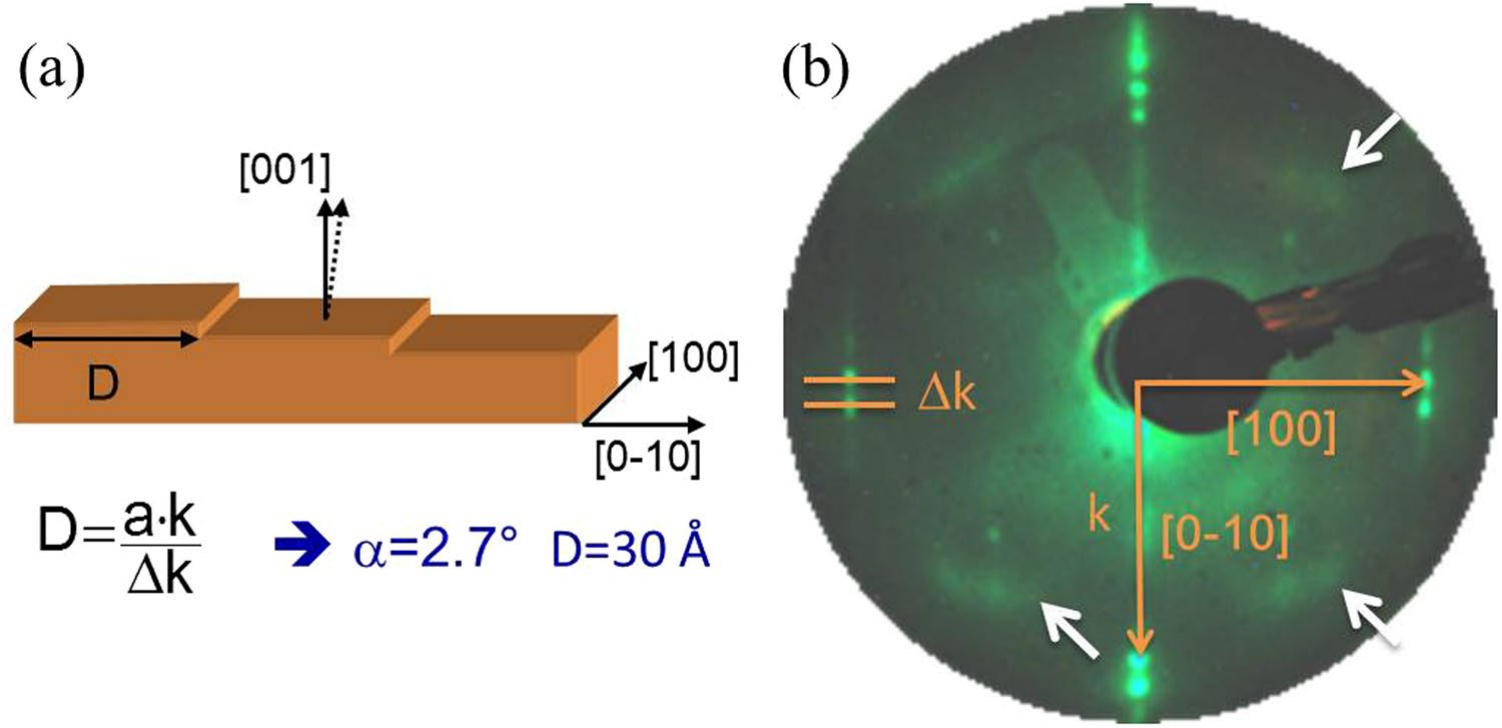
(**a**) Schematic representation of the Fe(0 18 1) surface with a miscut angle of 3° and steps running along the [0 ±1 0] direction. (**b**) Typical LEED picture of the C:c(3 $\surd$2 × $\surd$2):Fe(0 18 1) surface measured at 50 eV. At this electron energy the surface sensitivity is the highest. Two distinct features are observed: i) intense spots from the (0 0 1) surface split by presence of the steps along the [0 ±1 0] directions, ii) weak spots at c(2 × 2) positions surrounded by faint halos at the positions of the c(3$\surd$2 × $\surd$2) reconstruction, indicated by white arrows. From the splitting of the (0 0 1) spots an average terrace size of D = 30 Å has been obtained.

The LEED pattern of the as prepared surface is displayed in Fig. 1(b). It shows well defined intense spots split along the [0 ±1 0] directions, indicating a regular array of steps aligned along the [1 0 0] direction. The average width D of the terraces between the mono-atomic step edges was estimated from the measured splitting to be *D*~30 Å. This value corresponds to a miscut angle of 2.7°, in agreement with the expected value. Besides the main spots faint spots at (1/2, 1/2) fractional beams positions and weak halos around them can be clearly distinguished [white arrows in Fig. 1(b)]. These features, previously observed on flat Fe(0 0 1), indicate a c(3$\surd$2 × $\surd$2) reconstruction of segregated carbon atoms forming chains^11^.

**Figure 2:**
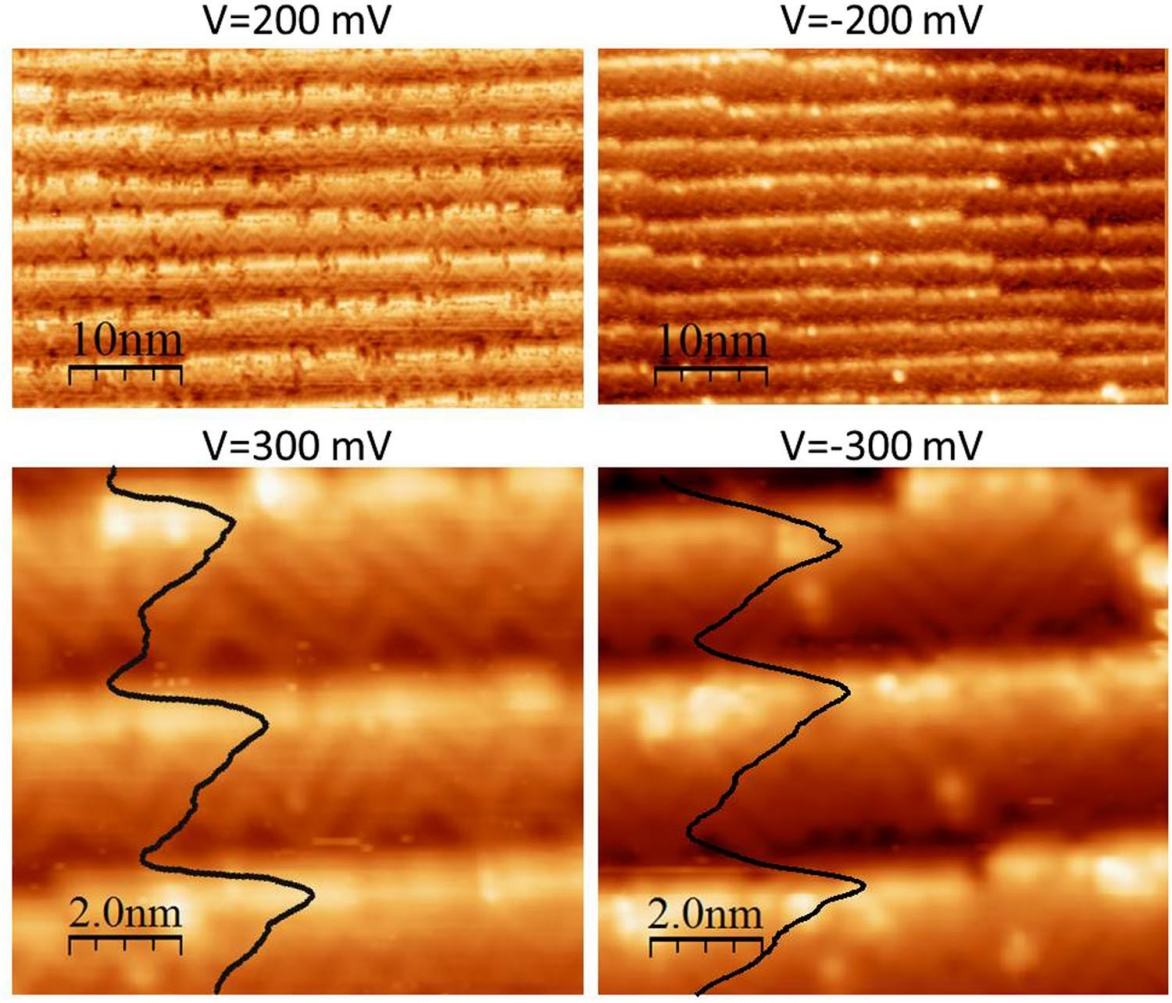
STM images over a large spatial range (top panels) and zoom outs representative of the prepared surface (bottom panels). The left panels were measured with V = 200 mV and V = 300 mV thus sampling the unoccupied states whereas the right panels were measured with V = −200 mV and V = −300 mV and sample the occupied states. Large brightness contrast can be observed at the terraces and high intensity at the step-edges. From the zoom out we can conclude the presence of chains in both empty and occupied electronic states. The black lines are intensity profiles showing maximum brightness at the step edges.

Indeed, STM measurements on Fe(0 0 1) have shown that for the c(3$\surd$2 × $\surd$2) reconstruction C atoms arrange in two row zig-zag chains running along the [±1 ±1 0] in-plane directions for a coverage of ~2/3 of a monolayer. The maximum chain length is ~20.0 Å and afterwards interruption or rotation by 90° occurs^11, 20^. We measured STM images on our stepped surface for both empty and occupied states. The results are displayed in Fig. 2 (left panel) for the empty and in Fig. 2 (right panel) for the occupied states. The upper images were taken with V = 200 mV and V = −200 mV in a large view. In both image types bright intensity -a factor of 3 higher than the average value- at the step edges and intensity variations on the terraces can be clearly observed. They correspond to a variation of the density of empty states (DOS). A zoom out with better resolution, Fig. 2 (bottom panel), allows to clearly disentangle the presence of chains on the terraces for the unoccupied states, bottom left panel of Fig. 2, as previously reported for the flat surface. The C atoms, occupying a slightly displaced hollow site position, attract towards them the Fe atoms contained within the two C row chains and form a one dimensional empty SS of Fe-${d}_{{z}^{2}}$ character, consistently with predictions from DFT calculations^21^. The bright areas correspond indeed to the density of Fe atoms within the rows of C atoms. Following this understanding of the flat surface we can explain the high intensity at the step edges by assuming the presence of Fe atoms within the carbon zig-zag chains. The image of the occupied states, Fig. 2 (bottom right panel), shows a similar intensity pattern as for the unoccupied states. We can therefore conclude that the chains can also be observed in the DOS of the occupied states. They are expected to have different Fe orbital character. The 1D chains run randomly along the [±1 ±1 0] directions, thus are rotated (±45°) with respect to the [0 −1 0] direction of the step edges and vary in length. Since the terrace size is 30 Å, larger than the average chain length observed on the flat surface (20 Å), the chains display the same random pattern as observed on the flat surface. More regular chains arrays are to be expected for stepped surfaces with miscut angles (>5.85°). In this case the average step size along the diagonals would match the average chain length. For steps running along the [±1 1 0] directions the chains would run perpendicular or parallel to the steps.

**Figure 3:**
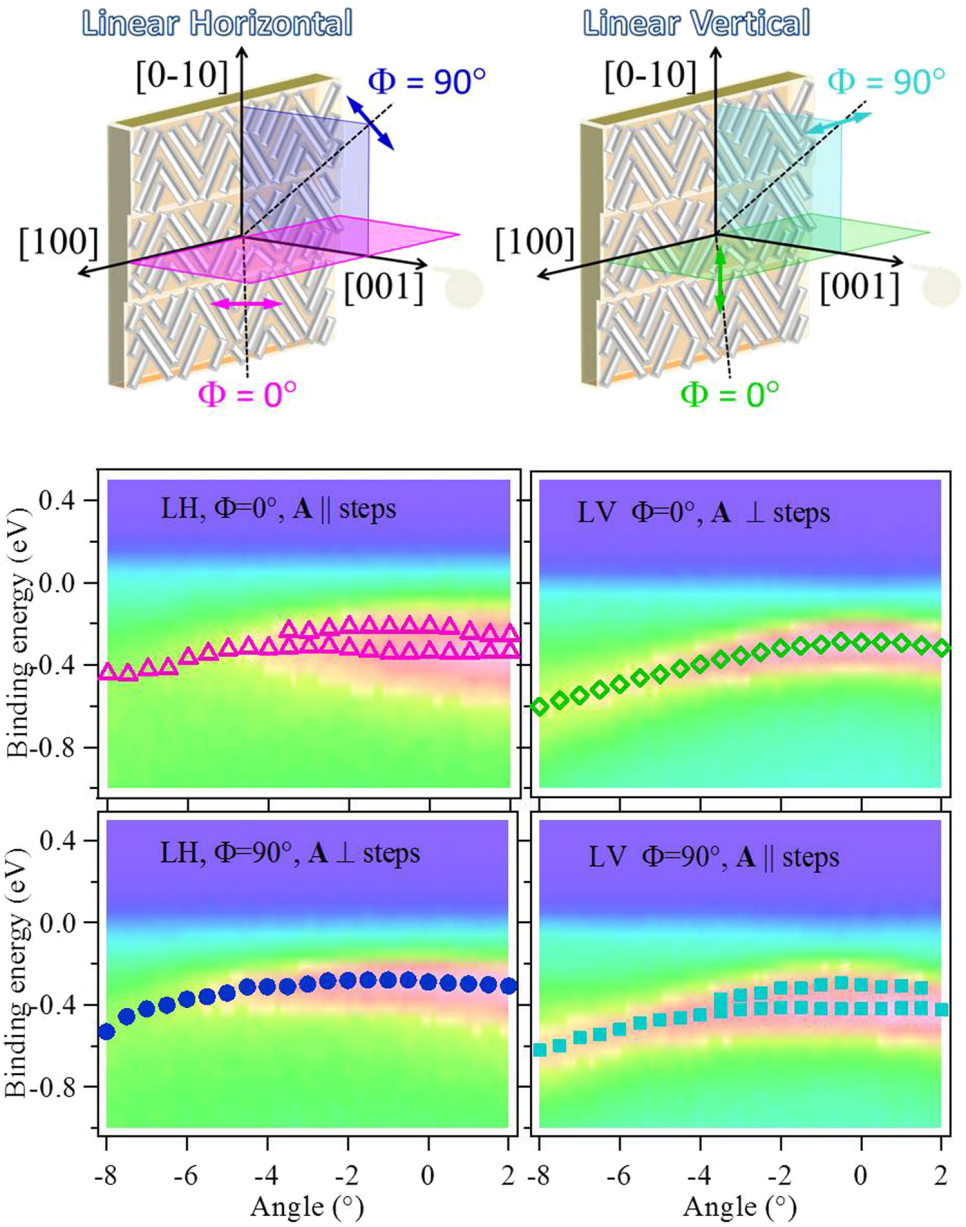
(Top panel) Geometrical configurations used in the experiment. (Bottom panel) ARPES intensity maps measured along the [1 0 0] and [0 −1 0] directions for LH and LV polarizations. The symbols on the images correspond to the intensity maxima measured for each angle and correspond to the energy dispersion of the states. Two geometries were realized: Φ = (0° ≡ 180°) and Φ = (90° ≡ 270°). For equivalent geometries only tiny differences in intensity are observed.

We performed high resolution ARPES experiments at room temperature using horizontal (LH) and vertical (LV) linearly polarized synchrotron radiation at the APE Low Energy beamline of Elettra (Trieste). The sample was oriented with the [0 0 1] high symmetry direction collinear with the normal emission (NE) direction and the axis of the photoelectron energy and momentum analyzer. In this geometry, the band dispersion along the [±1 0 0] and [0 ±1 0] surface high symmetry directions was measured in a large angular range with both LH and LV polarizations (Fig. 3 (top panel)). The bulk band dispersion (not shown) matches that of the standard Fe(0 0 1) surface. However, relevant differences with respect to the flat surface were observed in the behavior of the SS. In the following we will concentrate in the states close to the Fermi level.

**Figure 4:**
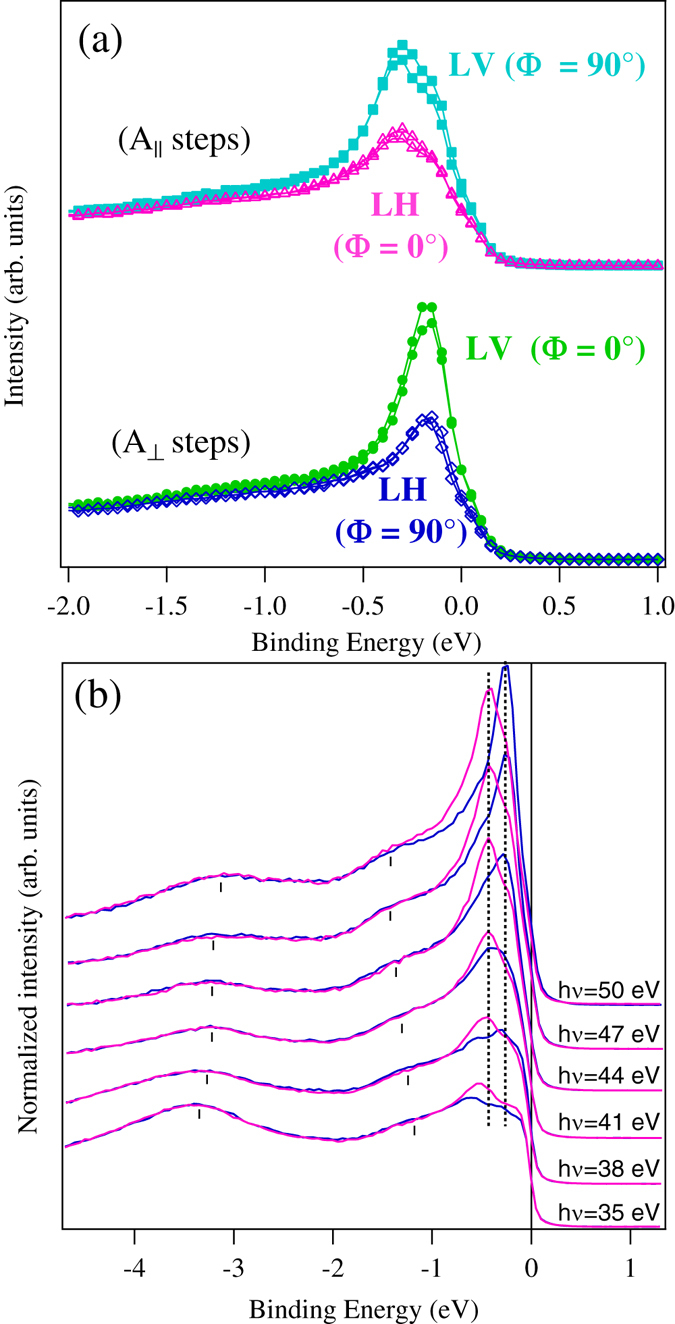
(**a**) EDCs measured in normal emission for LH and LV polarizations. The measurements show the presence of two states when **A** is parallel to the steps and one when it is perpendicular (see text for details). (**b**) Photon energy dependence of the EDCs measure with LH polarization along the [1 0 0] (Φ = (0°)) and [0 −1 0] (Φ = (90°)) directions in the range from 50 eV to 35 eV for LH. The steeply increasing intensity in this photon energy range uniquely identifies the *d* origin of the states^35^. The constant kinetic energy as a function of the photon energy confirms its surface state character. Below 40 eV they decay into surface resonances.

In the bottom panel of Fig. 3 intensity maps around NE measured at 50 eV photon energy are plotted. The dispersion of the bands close to the Fermi energy is indicated by markers. We can see that depending on the experimental geometry, e.g. as a function of the synchrotron light polarization one or two surface states are observed. The energy distribution curves (EDCs) angle integrated ±0.25° around NE with LH and LV polarization are displayed in Fig. 4(a). The EDCs obtained from the band dispersion along the [1 0 0] direction (*k*_||_ parallel to the steps) correspond to an azimuthal angle Φ = (0° ≡ 180°) and that along the [0 −1 0] direction (*k*_||_ perpendicular to the steps) to the Φ = (90° ≡ 270°). A double peak structure close to the Fermi level is well distinct in the data measured with LH (Φ = 0°) while a single peak dominates the spectra measured in the Φ = 90° configuration. A reversed behavior can be observed for LV polarization: the double peak appears for the (Φ = 90°) configuration and the single one for (Φ = 0°). The splitting of the surface state measured for LH-(Φ = 0°) and LV-(Φ = 90°) is consistent with the carbon reconstruction on flat Fe(0 0 1). Furthermore, the lineshape of the EDCs measured with LH-(Φ = 90°) and LV-(Φ = 0°) for which only one state can be observed is more similar to that measured for clean Fe(0 0 1) at the same photon energy (see Fig. 1 supplement material and ref. 20).

In order to confirm the surface state character of the states, measurements with LH (Φ = 0°) and (Φ = 90°) have been carried out as a function of the photon energy in the range from 50 eV to 35 eV. The results, displayed in Fig. 4(b) show that above 40 eV we can see the peaks close to the Fermi energy do not disperse with photon energy, confirming the surface character for both states. Below 40 eV the shape of the spectra changes substantially, the *d*-like surface state peaks loose intensity due to lower photoionization cross section -a factor of 2 reduction at h *v* = 35 eV with respect to h *v* = 50 eV- and the surface states overlap with surface resonances.

The azimuthal angle dependence of the double peak is unexpected since in the experimental configuration used the two azimuths are equivalent for each polarization. Therefore, the same dispersion of the electronic states was expected. Moreover, we observe that the spectral shape for LH (Φ = 0°) and LV (Φ = 90°) is the same except for the higher relative intensity for LV photons. A single state close to the Fermi energy can be observed for LH (Φ = 90°) and LV (Φ = 0°). This can be explained by the geometrical configurations used for LH and LV (Fig. 3, upper panel). In fact, the vector potential (**A**) of LV light lies on the surface and it is perpendicular(parallel) to the steps for Φ = 0°(Φ = 90°). The LH polarized light can be decomposed into one component parallel to the surface and another perpendicular to it. The intensity of each component is equally distributed in both cases due to the 45° incidence angle of the experiment. In this case the in-plane **A** component is parallel(perpendicular) to the steps for Φ = 0°(Φ = 90°). This 90° rotation explains the fact that the double(single) peaks are observed for different azimuths with LV and LH polarizations. Since these states are symmetric with respect to **A** for Φ = 0° and Φ = 90°, the observation of a double(single) peak dispersion depending on whether **A** is parallel(perpendicular) to the steps must the consequence of the existence of steps at the surface.

**Figure 5:**
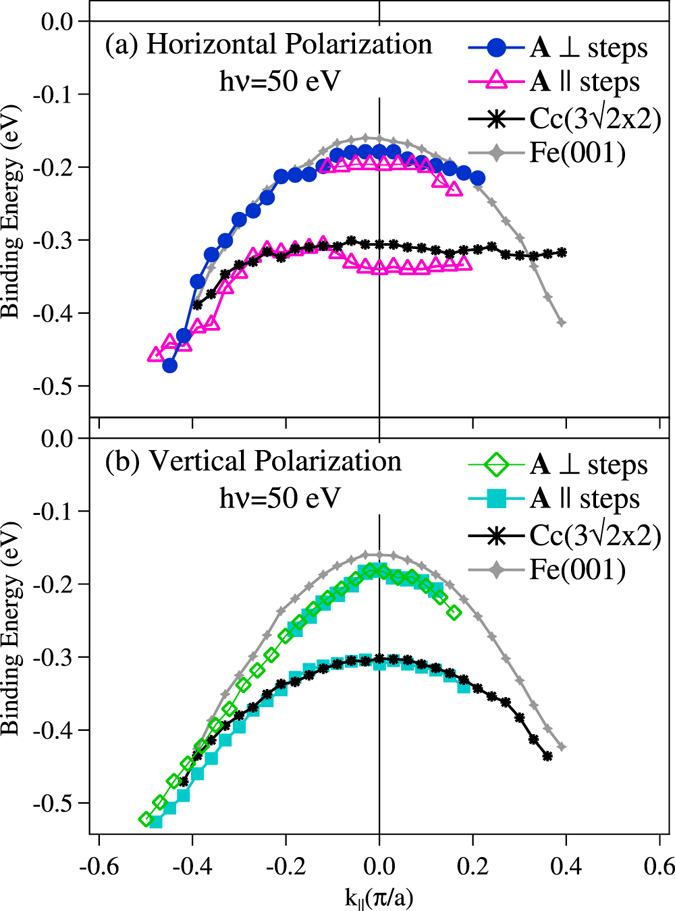
Dispersion of the electronic SS with *k*_||_ measured for the two polarizations and equivalent geometries of Fig. 3 plotted together with the dispersion measured for a flat clean Fe(0 0 1) surface (gray cross-marked line) and the C-c(3$\surd$2 × $\surd$2):Fe(0 0 1) (black start-marked line). The state close to the Fermi level in the stepped surface exhibits the same dispersion as that of clean Fe(0 0 1), the other disperses as the SS of the C-c(3$\surd$2 × $\surd$2) reconstructed surface. The latter state is only seen when **A** is parallel to the step edges indicating that it is confined on the terraces. The intensity of the Fe-like is enhanced when **A** is perpendicular, suggesting that the state is more localized at the step edges.

The band dispersion along the [1 0 0] and [0 −1 0], extracted from the intensity maps of Fig. 3 (bottom panel) for LH and LV polarizations, are displayed in Fig. 5. The dispersion of the two states observed when **A** is parallel to the steps (LH-Φ = 90°, LV-Φ = 0°) is different, with the high binding energy peak having a flatter *k*_||_ dispersion. The single state measured with **A** perpendicular to the steps (LH-Φ = 90°, LV-Φ = 0°) disperses and has the same binding energy as the low binding energy state measured for the other azimuth, thus pointing to a common origin. States seen with both LH and LV polarization have mixed symmetry. Slight differences in the dispersion for the two polarizations suggests interaction with bulk states^20^. The data for the C(c(3$\surd$2 × $\surd$2)) Fe(0 0 1) surface show only the high binding energy state mixture with both polarizations. It remains to understand the origin of the low binding energy component of the SS. To this end, the dispersion of a clean Fe(0 0 1) surface is also plotted. Surprisingly, the low binding energy state of the stepped surface disperses as the SS of the clean Fe(0 0 1). These results suggest that the C terminated stepped surface preserves the electronic and magnetic properties of clean Fe. In spite of the chain like behavior observed in STM no signatures of 1D SS confinement were observed at odd with the 1D SS confinement observed for Shockley states of the (1 1 1) terraces of vicinal surfaces of fcc noble metal and Ni^7, 32–34^.

## Discussion

From the band dispersion of the Fe(0 0 1) and carbon reconstructed surface, the nature of the 3 *d* orbitals can be disentangled. Close to the Fermi edge the occupied states can have *d*_*xy*_, *d*_*xz*_, *d*_*yz*_ character. The *d* character of the photoemission peak is clearly reflected in the twofold increase of photoionization cross section between h *v* = 35 eV and h *v* = 50 eV that is a signature of the 3d subshell^35^. The ${d}_{{z}^{2}}$ are empty^20, 21, 25^ and the ${d}_{{x}^{2}-{y}^{2}}$ surface states appear at much higher binding energy^25^. According to the dipole approximation the *d*_*xy*_ states are odd with respect to planes containing the **y**(**x**) vectors, thus for both emission planes in our geometry (Fig. 3, lower panel) and therefore can only be excited with LV polarization. The *d*_*xz*_(*d*_*yz*_) are even with respect to a plane containing the **x**(**y**) vector and can be excited with LH light when the latter has an **A**_*x*_(**A**_*y*_) component. On the opposite, the states are odd with respect to a plane containing the **y**(**x**) vector and therefore are observed with LV polarized light having **A**_*y*_(**A**_*x*_) components. Our experiments with LH polarization probe the *d*_*xz*_(*d*_*yz*_) for Φ = 0°(Φ = 90°) and with LV polarization both the *d*_*xy*_ and *d*_*yz*_ for Φ = 0°. For Φ = 90° the *d*_*xy*_ and *d*_*xz*_ orbitals are measured. The fact that for LH(LV) and Φ = 90°(Φ = 0°) only one state is observed could be interpreted with a high binding energy state of pure *d*_*xy*_ character, whereas the low binding energy state should have *d*_*xy*_ + *d*_*xz*_ + *d*_*yz*_ character. This orbital assignment is questioned by calculations on the reconstructed C-c(3$\surd$2 × $\surd$2):Fe surface showing the chains to have electron states derived from Fe_1_3*d*_*xz*_ + Fe_2_3*d*_*xz*+*xy*_ and Fe_1_3*d*_*xz*_ + Fe_2_3*d*_*xz*+*xy*_ orbitals for chains running at 90° with respect to each other^36^. When this orbital composition is considered, intensity coming from the *d*_*xy*_ and *d*_*yz*_ orbitals should show up for the LV-Φ = 0° configuration. Similarly *d*_*yz*_ contribution should be observed for LH-Φ = 90°, which is not the case.

The results for the low binding energy state are compatible with a mixture of *d*_*xz*_ + *d*_*yz*_ as expected for clean Fe^25^. The fact that only one state is seen when the potential vector is perpendicular to the steps suggests that Fe-like states are located at the step-edges and the c(3 $\surd$2 × $\surd$2) on the terraces. For **A** perpendicular to the steps photoexcitation of states at the step-edges will be then favored. This interpretation is supported by the STM measurements showing a higher intensity at the step edges compared to the terraces as seen in Fig. 2 (bottom panel). This enhanced intensity has been observed on monoatomic steps on clean Fe and interpreted as enhanced scattering at the step edges^6^. We rather connect the enhanced empty state intensity as probed by STM to the presence of Fe-like surface states at the step edges, as demonstrated by its distinct band dispersion. This mixed electronic properties have been observed in other systems, like Au:Si(5 5 3) where Au chains are located on the terraces and the step-edges have a Si honey-comb structure with spin-split surface states^3^.

**Figure 6:**
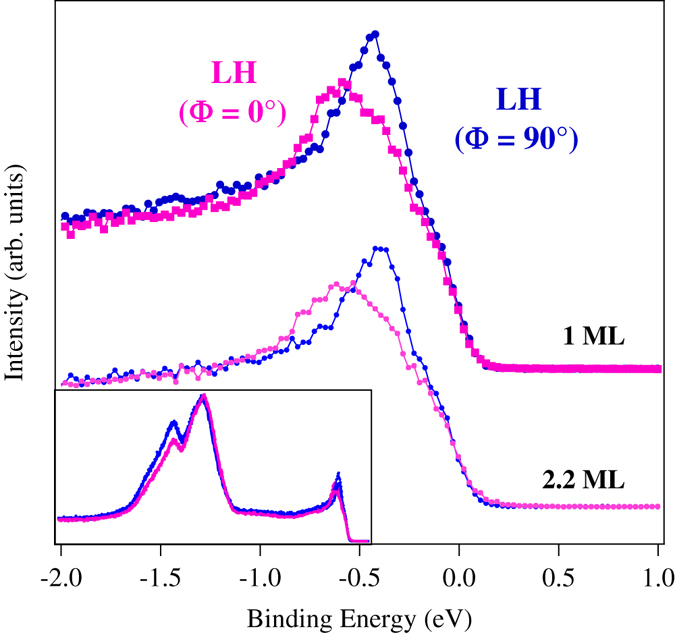
EDCs measured after deposition of 1 and 2.2 layers of Ag on C-c(3$\surd$2 × $\surd$2):Fe. The Blue(magenta) curve corresponds to the measurements with **A** perpendicular(parallel) to the steps. The same SS as in 4 are present, confirming the passivation effect of the carbon reconstructed surface. The inset shows the EDC energy range measured [−14, 1] eV showing the Ag valence band.

The C:c(3 $\surd$2 × $\surd$2) reconstruction has shown to passivate the Fe(0 0 1) surface^20^. In the present case we observe not just passivation but stabilization of the surface electronic states of the clean Fe surface. The robustness of the passivation was tested by depositing Ag on top of the C:c(3 $\surd$2 × $\surd$2):Fe(0 18 1) at room temperature. The EDCs displayed in Fig. 6 show that the splitting of the states at normal emission is present even after full coverage of the surface by *in situ* deposition of 2.2 monolayers of Ag -Deposition of Ag has been chosen due to the well known growth of Ag on Fe(0 0 1)^37, 38^. This interface has been extensively studied after the observation of quantum confinement effects^39, 40^. Metal coverage of stepped surfaces generally uptakes from the step edges. The permanence of the Fe surface state signature indicates that the surface electronic structure of the present system is indeed effectively protected^2, 3^. No signature of quantum confinement was observed. This is different from other transition metal stepped surfaces where confinement of surface states in the direction perpendicular to the terrace edges has been observed^32–34^. In the present case the apparent lack of 1D confinement can be understood in connection with the interaction between the cross-linked chains, at 45-degrees with respect to the step edges, that determine a 2D network of surface states. The energy position of the states after Ag deposition shifted to 0.137 eV higher binding energies. The presence of the photoemission surface peaks after Ag deposition confirms the passivation role of the carbon termination and supports the interaction of the surface states with bulk states. The effective protection of the surface electronic structure of iron by carbon and by nanostructuring (steps at vicinal surfaces in the present case) indicates that suitable surface engineering could become relevant in, e.g. MTJ technology. The problems related to interface magnetically dead-layers, or with the loss of the surface-enhanced magnetic moments, are potentially overcome if a suitable surface nano-engineering is developed exploiting both structural, morphological and chemical protection. One very interesting possibility would be to realize Fe/MgO/Fe(0 18 1) junctions or Fe/Ag/Fe(0 18 1) spin valves to explore the possibility to enhance, by this architecture, the performance of these systems.

## Methods

The route used to produce the carbon terminated stepped surface was to start from a Fe single crystal with bulk C impurities. By a combination of Ar-ion sputtering cycles at temperatures between 570 K and 670 K followed by flashes at 875 K the desired surface was obtained. Other routes like evaporation of carbon on a pristine Fe(0 18 1) surface followed by annealing procedures are expected to give similar results.

To characterize the properties of the obtained surface we used three different techniques: LEED (low energy electron diffraction), STM (scanning tunneling microscopy) and ARPES (angle resolved photoemission spectroscopy). All experiments were performed *in-situ* at the APE-LE beamline of IOM-CNR at Elettra Sincrotrone-Trieste. The ARPES experiments were done using linearly polarized undulator radiation in the 10–100 eV energy range. The total energy resolution of the SCIENTA-2002 analyser and the monochromator was kept around the thermal broadening value, *k*_*B*_ · T ~ 25 meV. The STM data were measured at room temperature. Typical currents between 100 to −550 pA were used for the images measured with negative voltage. For the positive voltage images the currents used were in the 700 pA to −350 pA.

## Electronic supplementary material

Supplementary information
