# Supplementary material for: Protected surface state in stepped Fe (0 18 1)
Source: Sci Rep. 2017 Jul 26;7:6609. doi: 10.1038/s41598-017-06896-4 (PMC5529575; doi:10.1038/s41598-017-06896-4)
Supplement: Supplementary file 1 — Supplementary information [file 41598_2017_6896_MOESM1_ESM.pdf]

Protected surface state in stepped Fe (0 18 1)

Manuel Izquierdo[1,2,*], Piero Torelli[3], Jun Fujii[3], Giancarlo Panaccione[3], Ivana Vobornik[3], Giorgio Rossi[3,4], and Fausto Sirotti[2,5]

[1]European XFEL Gmbh, Albert-Einstein-Ring 19, 22761 Hamburg, Germany
[2]Synchrotron Soleil, L'Orme des Merisiers St Aubin, BP 48, 91192 Gif-sur-Yvette, France
[3]CNR-IOM, TASC Laboratory, in Area Science Park, S.S.14, Km 163.5, I-34149 Trieste, Italy
[4]Dipartimento di Fisica, Università di Milano, via Celoria 16, 20133 Milano, Italy
[5]Physique de la Matiére Condensé UMR 7643, CNRS and Ecole Polytechnique, Université Paris Saclay 91128 Palaiseau, France
[*]manuel.izquierdo@xfel.eu

Supplement material

In figure 1 the intensity maps for the C:c(3$\sqrt{2}$x$\sqrt{2}$):Fe(0 0 1) are displayed. Only one intensity maxima can be clearly disentangled, at odd with our C:c(3$\sqrt{2}$x$\sqrt{2}$):Fe(0 18 1) surface.

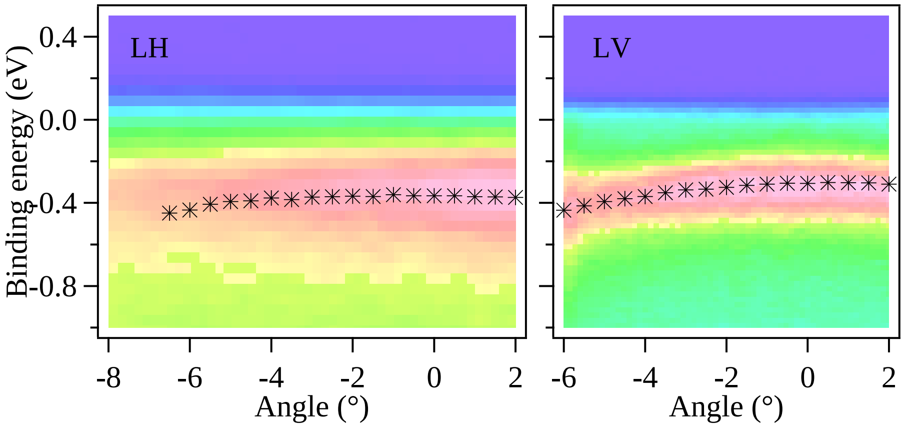

Figure 1. (Color online) ARPES intensity maps measured along the [1 0 0] directions for LH and LV polarizations for the C:c(3$\sqrt{2}$x$\sqrt{2}$):Fe(0 0 1) terminated surface. The dark makers indicate the position of the intensity maxima. We can see that only one peak can be observed for each polarization.

In order to verify that the electronic structure of the surface under investigation has the electronic properties of both the clean Fe(0 0 1) and C:c(3$\sqrt{2}$x$\sqrt{2}$):Fe(0 0 1) surfaces we have plotted in figure 1 EDCs measured at NE with LH (2(a)) and LV (2(b)) for our C:c(3$\sqrt{2}$x$\sqrt{2}$):Fe(0 18 1) termination for two azimuths together with those measured for the Fe(0 0 1) and C:c(3$\sqrt{2}$x$\sqrt{2}$):Fe(0 0 1). We can observe two states on the C:c(3$\sqrt{2}$x$\sqrt{2}$):Fe(0 18 1) terminated surface while only one is observed for the flat surfaces. The low binding energy corresponds to the C:c(3$\sqrt{2}$x$\sqrt{2}$):Fe(0 0 1) terminated surface and the high binding energy to the Fe(0 0 1) flat surface. The presence of a single surface state in the flat surfaces and a double in our C:c(3$\sqrt{2}$x$\sqrt{2}$):Fe(0 18 1) terminated surface is independent of the light polarization. Therefore we can conclude that our observation is an intrinsic property of the surface and not related to the photoemission process.

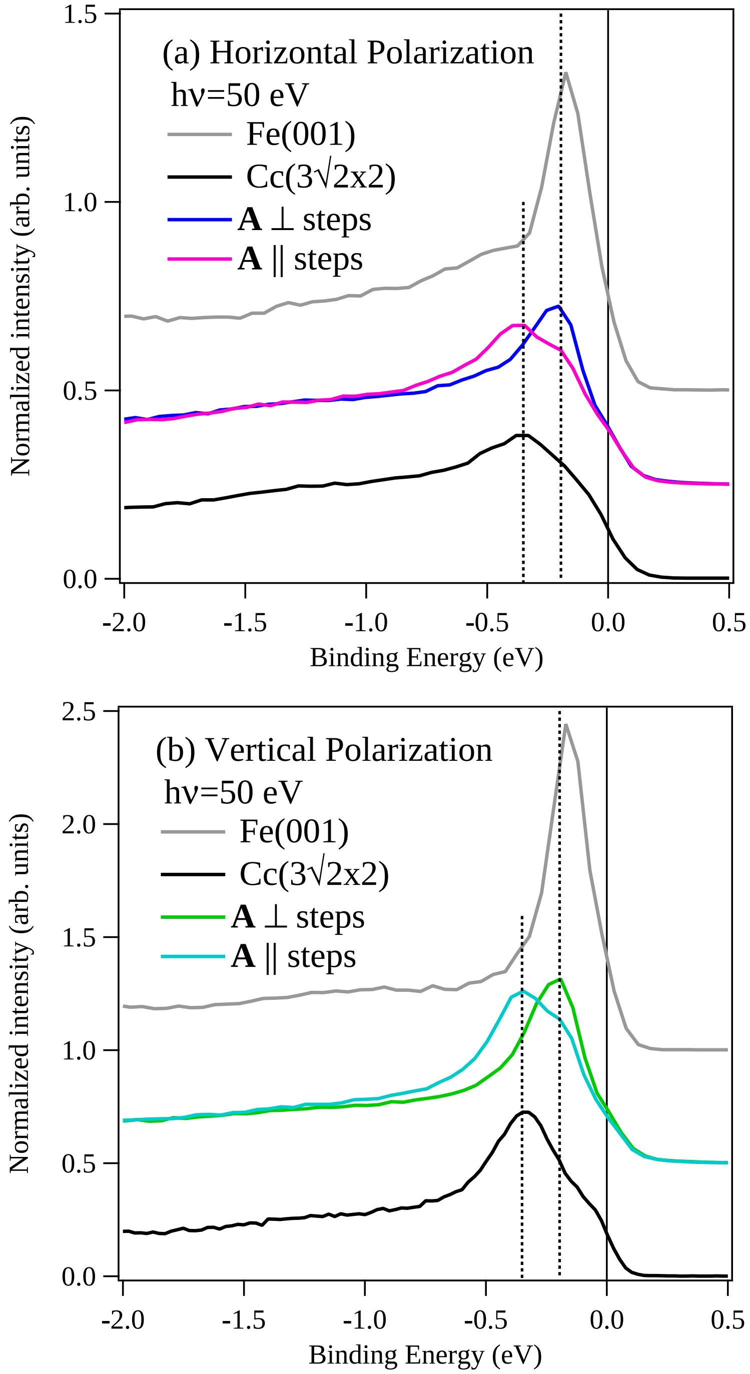

Figure 2. (Color online) (a) EDCs measured in normal emission with LH for the Fe(0 0 1) (gray), C:c(3$\sqrt{2}$x$\sqrt{2}$):Fe(0 0 1) (black) and the C:c(3$\sqrt{2}$x$\sqrt{2}$):Fe(0 18 1) with $A_{||}$ (magenta) and $A_{\perp}$ (blue). (b) EDCs measured with LV photons for the C:c(3$\sqrt{2}$x$\sqrt{2}$):Fe(0 18 1) with $A_{||}$ (light blue) and $A_{\perp}$ (green). For the flat surface the same color code as in (a) was used. We can see that the EDCs of the stepped surface has two peaks when the potential vector is parallel to the step edges and only one when it is perpendicular to them. The electronic properties of the stepped surface seems to be an average of the two flat surfaces. The higher intensity measured for the Fe(0 0 1) surface is due to the lower temperature used in the measurements.
